# Heated Air

**Published:** 1906-04

**Authors:** J. Zederbaum


### HEATED AIR.

I wish to say a few words in favor of heated air. I have yet to meet a patient who did not find immediate relief while the preparation of some sensitive cavity was done under dry conditions brought about by heated air pressure. Warm air for obtunding hypersensitiveness of dentine was used for many years; but not until the last few years did it become as effective and so generally used; and simply because we never had a continuous strong stream of air until we get to using compressed air outfits. We also, have better ways of hearting the air; and we run less danger of burning the soft tissues, because we need not have the nozzle in the cavity; a few inches away will do better, and the air coming as it does in one continuous stream does not get cooled enough before reaching the cavity to retard the desired effect. One word about the manner of using: One should never attempt to employ the compressed air at a higher temperature than can be comfortably borne on the back of ones hand. Always start in slowly and do not direct the current immediately at the point to be operated upon; but a little away at first, closing in gradually until you are sure you have accustomed the tissues to its influence and have dispelled your patient's possible fright and thereby perhaps avoided some disastrous result. Here we must be even more useful then when soldering a porcelain bridge—here, we deal with a live subject capable of responding even with fistic demonstrations.

Other invaluable uses of compressed air in dentistry are: Its application in pyropylactic treatment, in cleansing the teeth, and in treating pyorrhoeal pockets. This is a prophylactic age, and here, there and everywhere we hear of prophylactic measures calculated toward bettering the general conditions in mouths. I regard a thorough cleansing of teeth and massage of gums as one of the most important operations we are called upon to perform (and I charge accordingly).—*Dr. J. Zederbaum, Extract Odontoblast.*

